# Successful Transmission and Isolation of a Fungal Pathogen From Wild Frogs to a Captive Amphibian Model Species: Fine Scale Pathogen Genetic Diversity and Infection‐Induced Changes in Skin Bacteria

**DOI:** 10.1111/1462-2920.70136

**Published:** 2025-07-03

**Authors:** Tamilie Carvalho, Daniel Medina, Timothy Y. James

**Affiliations:** ^1^ Department of Ecology and Evolutionary Biology University of Michigan Ann Arbor Michigan USA; ^2^ Institute for Global Change Biology School for Environment and Sustainability, University of Michigan Ann Arbor Michigan USA; ^3^ Center for Tropical Island Biodiversity Studies The School for Field Studies Isla Colón Bocas del Toro Panama

**Keywords:** amphibian pathogen, *Batrachochytrium dendrobatidis*, Bd–host–microbiome interactions, *Hymenochirus boettgeri*, transmission‐experiment

## Abstract

Amphibian populations worldwide are severely threatened, in part, by the pathogenic fungus *Batrachochytrium dendrobatidis* (Bd). While Bd has driven many amphibian declines and extinctions, its impact varies, with some populations exhibiting resistance or tolerance. Understanding the mechanisms behind this variation, together with Bd genetic diversity, is crucial for conservation. We used the model organism *Hymenochirus boettgeri* in a lab‐based transmission experiment designed to isolate Bd derived from wild amphibian hosts with low pathogen loads. Through successful transmission and subsequent isolation and genotyping, we identified multiple Bd genotypes from a single population, including a potential sexual recombinant, all belonging to the Global Panzootic Lineage (Bd‐GPL). This finding contributes to evidence of ongoing genetic mixing of Bd in natural environments. Additionally, we leveraged this experiment to assess Bd‐induced changes in amphibian skin bacteria. Our results showed significant changes in the skin bacterial communities of *H. boettgeri* after acquiring Bd infection, including reductions in bacterial diversity, and shifts in community composition, as observed in others susceptible species. These findings highlight the value of transmission experiments in isolating Bd from wild hosts and underscore the potential of *H. boettgeri* as a model to investigate Bd genetic diversity and host‐associated microbiome responses to infection.

## Graphical abstract

The isolation of *Batrachochytrium dendrobatidis* (Bd) from low‐load wild amphibians revealed multiple genotypes, including a potential hybrid, highlighting ongoing pathogen genetic mixing in natural environments. Additionally, Bd infection induced skin microbiome shifts in *Hymenochirus boettgeri* , demonstrating its utility as a model for studying pathogen–microbiome interactions.

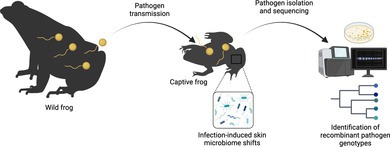

## 1 Introduction

Amphibians are facing an unprecedented biodiversity crisis, with more species threatened with extinction than any other vertebrate group (IUCN 2024). A key driver of this crisis is the fungal pathogen *Batrachochytrium dendrobatidis* (Bd), the cause of chytridiomycosis, which has led to dramatic declines and extinctions of amphibian species across the globe (Scheele et al. 2019; Luedtke et al. 2023). Bd is a generalist pathogen that infects the keratinized skin of hundreds of species of amphibians, disrupting epidermal function and causing a series of electrolyte imbalances, which can ultimately lead to cardiac failure and death (Voyles et al. 2011). However, variation in susceptibility to Bd is extreme among hosts, with some highly susceptible species facing extinction while others show significant resistance or tolerance (Carvalho et al. 2024). The factors driving this variation in susceptibility are not fully understood, though evidence suggests that host immunogenetic variation, life history traits, and differences in skin microbiomes are associated with such variation (Fisher and Garner 2020; Zamudio et al. 2020; Carvalho et al. 2024).

Variation in susceptibility is influenced not only by host factors but also by the pathogen's genetic characteristics. Bd is genetically diverse, and this diversity plays a crucial role in determining its virulence across host species (Fisher and Garner 2020; Fisher et al. 2021). Isolation and sequencing of the pathogen have revealed multiple distinct lineages and genotypes, including the hypervirulent global panzootic lineage (Bd‐GPL), which has been associated with most amphibian declines globally (Farrer et al. 2011; O'Hanlon et al. 2018; Scheele et al. 2019). Conversely, geographically restricted endemic lineages typically exhibit a reduced degree of virulence or competitiveness in their hosts, presumably due to a long evolutionary history of coexistence with their hosts (Jenkinson et al. 2018; Belasen et al. 2022; Carvalho, Medina, et al. 2023; Carvalho, Si, et al. 2023). Yet, GPL is present in populations where no declines due to chytridiomycosis have occurred or been detected, such as the southeastern USA, the UK, and Asia (Scheele et al. 2019; Carvalho et al. 2024). Adding complexity to this scenario, Bd‐GPL is hybridising with local endemic lineages, and hybrid genotypes have shown increased virulence in certain species (Greenspan et al. 2018; Carvalho, Medina, et al. 2023; Carvalho, Si, et al. 2023). Recent evidence also suggests recombination within the Bd‐GPL lineage (Byrne et al. 2022), which could potentially alter its pathogenicity and influence local host–pathogen dynamics. Such dynamic population genetic structure thus adds another layer of complexity to our ability to understand Bd's impact on amphibians. Nonetheless, significant knowledge gaps about Bd's global distribution and genetic diversity persist, hindering effective outbreak mitigation and conservation strategies.

While new genotyping methods allow the identification of Bd genotypes directly from tissue samples or skin swabs, they are costly and perform best with high infection loads (Byrne et al. 2017; Mulder et al. 2024). As a result, pure cultures remain essential for comprehensive genotyping. However, isolating Bd from low‐load infections remains a major challenge when using standard isolation methods, such as toe‐clipping from adults and infected tadpole mouthparts (Fisher et al. 2018). This has led to an imbalance in the global distribution of isolates, with gaps in critical regions that are essential for a better understanding of the pathogen's evolution (Carvalho et al. 2024). Moreover, the limitation in obtaining isolates from the wild restricts our ability to detect unknown lineages and conduct key experiments on virulence, transmission, and host–pathogen interactions, which are fundamental for understanding Bd's ecological dynamics and its impact on amphibian populations (Fisher and Garner 2020). Thus, despite advances in molecular genotyping methods, improving Bd isolation methods remains crucial for both ecological and experimental research.

Michigan, USA, serves as an example of a region where Bd has been present for many years but has never been isolated from wild hosts (Steiner and Lehtinen 2008; Zellmer et al. 2008; Zippel and Tabaka 2008; Igleski and Nicholson 2014; Talley et al. 2015). Despite infection loads being very low, suggesting disease endemism (Igleski and Nicholson 2014), the Northern cricket frog ( *Acris crepitans* ) is experiencing local declines (Steiner and Lehtinen 2008). These declines may be linked to Bd infection (Sonn et al. 2020; Wetsch et al. 2022), indicating a need for a better understanding of Bd's genetic structure in the region. It is possible, however, that endemic North American‐specific lineages of Bd may exist, especially since endemic lineages have been found on most continents (Carvalho et al. 2024). If present, these lineages could have lower virulence, which could explain low infection intensity and stimulate immune protection from more virulent genotypes.

Here, we conducted a transmission experiment using the amphibian model *Hymenochirus boettgeri* (Dwarf African frog), to capture and isolate Bd from wild amphibian populations where the pathogen load is low, using Michigan as a case study. The Dwarf African frog has recently been suggested as a model organism for studying Bd–amphibian interactions (Carvalho, Si, et al. 2023) and was chosen for its high susceptibility to Bd infection and extremely thin interdigital webbing, which facilitates the visualisation and isolation of the fungus. This approach allowed us to isolate Bd from wild Michigan populations for the first time.

Additionally, we leveraged this transmission experiment to assess whether pathogen infection was associated with changes in the skin bacterial communities of *H. boettgeri* , as observed in other Bd‐susceptible amphibian species (Rebollar et al. 2020). Host‐associated microbiomes play a crucial role in shaping pathogen dynamics, influencing both infection outcomes and host defence mechanisms (Flórez et al. 2015). In amphibians, skin microbial communities harbour a vast diversity of bacteria, some of which produce secondary metabolites capable of inhibiting the growth of Bd (Brucker et al. 2008; Becker et al. 2009). Understanding how Bd interacts with the amphibian microbiome is essential for uncovering host defence mechanisms and developing microbiome‐based conservation strategies (Walke and Belden 2016). Laboratory experiments have demonstrated that Bd can have mixed effects on amphibian skin microbiota. For instance, some species experience Bd‐induced reductions in skin bacterial diversity whereas other show no clear effect (Becker, Walke, Cikanek, et al. 2015; Walke et al. 2015; Longo and Zamudio 2017; Muletz‐Wolz et al. 2019; Hughey et al. 2022)—an outcome potentially associated with species Bd susceptibility and environmental conditions (Bernardo‐Cravo et al. 2020). Hence, these findings underscore the importance of using Bd‐susceptible species in controlled experiments to better understand how Bd impacts amphibian‐skin microbial communities, and the potential role of these bacteria, including some specific taxa, in mediating host resistance to infection. A microbiome response in *H. boettgeri* similar to that observed in threatened species would expand the use of this model organism for developing microbiome‐based interventions and treatments.

This experiment shows the successful transmission and isolation of Bd from wild amphibians to *H. boettgeri* , the identification of multiple Bd strains, including a potential recombinant genotype, and Bd‐induced changes in the skin bacterial community composition and structure. These results reinforce the use of *H. boettgeri* as a model organism in future investigations aiming to understand Bd infection dynamics, expand its application for isolating Bd and studying the role of the skin microbiome in pathogen defence, and highlight transmission experiments as a powerful approach to isolate Bd from wild amphibian populations, even in regions with low infection loads.

## 2 Methods

All procedures described here were conducted at the University of Michigan Life Sciences Institute Animal Care Facilities, following protocols approved by the University's Institutional Animal Care and Use Committee (IACUC protocol PRO00009614).

### 2.1 Transmission Experiment

#### 2.1.1 Transmission Assay

We sampled wild adult frogs on the night of May 26, 2022 from a natural pond at the Edwin S. George Reserve (ESGR), a 525‐ha ecological area in Livingston County, Michigan (42°26′44.3″ N, 84°00′57.0″ W), about 25 miles northwest of the University of Michigan campus in Ann Arbor. The collection was not targeted to any specific species; we sampled all frogs encountered at the site, including *Anaxyrus americanus, Rana clamitans, Rana pipiens*, and *Hyla versicolor* . We individually captured frogs using sterile plastic bags (Whirl‐Pak, Nasco, Fort Atkinson, WI, USA) and transported them to a field laboratory. We swabbed and handled each adult frog with a new pair of disposable gloves to avoid potential Bd cross‐contamination, and gently placed them into individual sterile containers, which were treated with 1% Virkon for 20 min and then rinsed with autoclaved water. We used DRYSWAB—Fine Tip Plastic Swab—Rayon Bub—Peel Pouch (MW113), manufactured by Medical Wire & Equipment, for sample collection. We kept frogs in these containers for ~15 h while samples were being processed to assess their Bd infection status. We transported the swabs on ice to the University of Michigan and stored them at −20 °C overnight. After DNA extraction and pathogen quantification (see details below), we selected four Bd positive wild frogs for the laboratory transmission experiment and released the remaining individuals at the collection site the following day.

**FIGURE 1:**
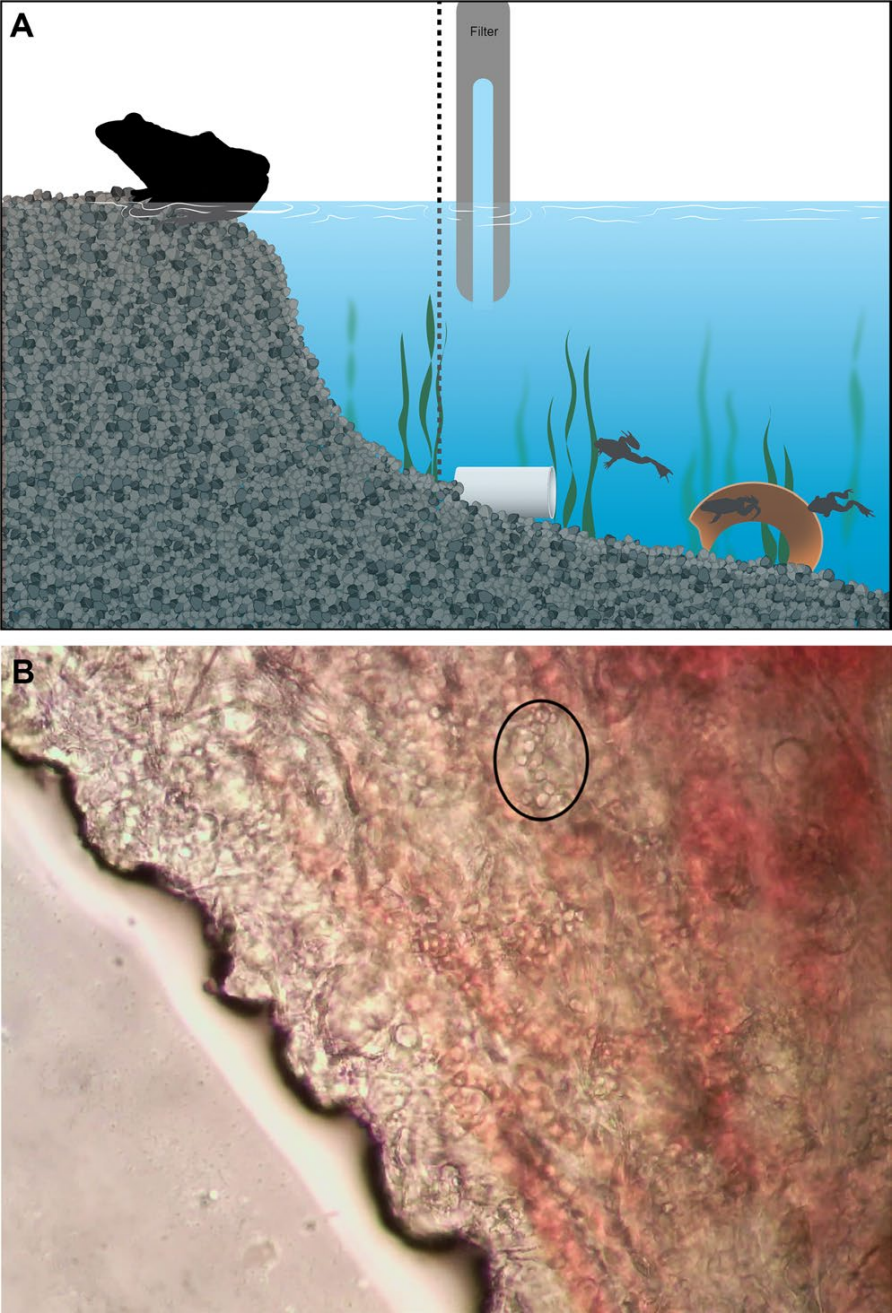
(A) Illustration of 38 L glass tank setup during transmission experiment, housing three *Hymenochirus boettgeri* and one wild frog. (B) *H. boettgeri* 's webbing skin infected with Bd. Black circle highlights Bd zoosporangia. Image taken under a microscope at 40×.

We set up four mixed‐species 38 L glass tanks (25 × 30 × 50 cm) with a tank divider (hole diameter of 1 mm) to prevent predation and allow only water circulation between both sides. We added 1 cm of gravel substrate to all tanks on the side of *H. boettgeri* frogs and enough gravel to make a dry space on the wild frog's side. Additionally, we added artificial plants, PVC pipes, and broken terracotta pots to each tank for retreat (Figure 1A). We sterilised all enrichment items using 1% Virkon for 20 min and then rinsed them abundantly with reverse osmosis (RO) water. We equipped tanks with an internal aquarium filter (Tetra, Spectrum Inc., Blacksburg, VA, USA) and covered them with plastic lids to prevent frogs from escaping. Lids contained small holes (of 1 cm of diameter) for air circulation. We filled the aquaria with RO water adjusted to a conductivity of 900 ± 100 μS/cm by adding 0.5 g/L of formulated synthetic sea salt mixture (Instant Ocean Sea Salt, Blacksburg, VA, USA). We conducted the experiment in a temperature‐controlled room set at 20 °C and a 13:11 light–dark cycle.

Each tank housed three individuals of *H. boettgeri* (6 months old and born in our facility; mean SVL = 2.16 ± 0.07 cm, *n* = 12; mean weight = 0.68 ± 0.07 g, *n* = 12) and either an American toad ( *A. americanus* ; Tank 1), a Green frog ( *R. clamitans* ; in duplicate, Tanks 2 and 3), or a Northern Leopard frog ( *R. pipiens* ; Tank 4). To assess Bd transmission and infection load throughout the experiment, we swabbed all live animals on days 10, 17, and 23 post‐Bd inoculation. We confirmed that all *H. boettgeri* were Bd‐free at the beginning of the experiment based on qPCR of skin swabs (see details below).

We fed frogs *ad libitum* three times a week with calcium‐enriched crickets for wild frogs and frozen gamma‐irradiated bloodworms for *H. boettgeri* . We tested water quality in all aquaria three times per week using an EcoTestr CTS1 meter (Oakton, Vernon Hills, IL, USA) for temperature (20°C ± 1°C) and conductivity (900 ± 100 μS/cm), and a water testing kit for pH (7 ± 0.2), ammonia (≤ 1.25 ppm), nitrite (≤ 0.25 ppm), and nitrate (≤ 10 ppm). We performed a 70% water change weekly and conducted all water changes on the same day for all aquaria. We monitored frogs four times daily throughout the experiment and euthanized animals if they showed a lack of righting response or at the end of the experiment. We euthanized the frogs using an overdose of tricaine methanesulfonate solution with pH adjusted to 7 (MS‐222; 0.5 g/L; Syndel, Ferndale, WA, USA).

#### 2.1.2 DNA Extraction and Quantification

To collect DNA from skin bacteria and Bd, we rinsed each animal with sterilised distilled water for 5 s and collected skin swabs following a standard protocol using disposable gloves (Hyatt et al. 2007). We extracted DNA from swabs using the DNeasy Blood and Tissue Kit (Qiagen, Valencia, CA, USA) following the manufacturer's protocol with a minor modification, which consisted of an extended incubation time (3 h) in the lysis step to increase DNA yield. We assessed Bd presence and intensity using a standard qPCR assay (Boyle et al. 2004) on a QuantStudio 3 Thermal Cycler (Applied Biosystems, Thermo Fisher Scientific). We generated standard curves using serial dilutions (10^6^–10^0^ zoospore equivalents, ZE) of the Bd strain JEL 423. We ran samples in duplicate to ensure accurate quantification, and only those samples that amplified ≥ 1 ZE in both replicates were considered positive for Bd. We repeated samples with inconsistent results and considered positive when two out of three replicates were positive. Infection loads represent the mean of ZE recorded for each positive replicate. We included negative controls in qPCR assays to account for contamination.

#### 2.1.3 Bd Isolation

To isolate Bd, we used sterile scalpels and forceps to collect skin tissue samples from the webbing between the toes of *H. boettgeri* after euthanasia or death. The thin nature of this skin tissue allowed regions infected with Bd to be easily identified under a microscope at 40× (Figure 1B). All *H. boettgeri* were used for isolation attempts following the protocol of Longcore (2000) for cleaning the skin (i.e., dragging the skin through an agar medium with a sterile needle to remove surface‐contaminating bacteria and fungi) with a few modifications: (i) We cleaned skin pieces on nutrient‐free agar plates with the addition of three antibiotics (200 mg/L penicillin‐G, 400 mg/L streptomycin sulfate, and 100 mg/L ciprofloxacin; all of them purchased from Thermo Fisher Scientific); (ii) after cleaning, we placed skin pieces on fresh plates of 1% tryptone agar (10 g tryptone, 10 g agar, and 1 L distilled water) with the same concentrations of the three antibiotics as described above; (iii) we incubated our plates at 10 °C to retard any growth of contaminants; and (iv) for stock cultures, we used 1% tryptone liquid (10 g tryptone and 1 L distilled water) without antibiotics.

If agar plates with Bd growth exhibited signs of contamination with fungal hyphae, we cut pieces of agar (1 cm × 1 cm) containing the skin using a clean spatula and transferred it to clean glass culture tube filled with 5 mL of 1% tryptone liquid with the same three antibiotics and concentrations. All tubes were closed and refrigerated at 5 °C. After 2 weeks, we carefully transferred approximately 600 μL of liquid culture without visible fungus hyphae to a fresh 1% tryptone agar plate with antibiotics. We incubated plates at 10 °C and inspected them daily. After 1 week, we transferred Bd colonies to a stock solution of 1% tryptone liquid without antibiotics.

#### 2.1.4 Whole‐Genome Sequencing of Bd Isolates

To obtain Bd DNA for whole‐genome sequencing (WGS), we transferred 600 μL of Bd stock solutions to 1% tryptone agar plates and allowed them to grow for 1 week at 21 °C. We extracted DNA from pure Bd cultures using the DNeasy Blood and Tissue Kit (Qiagen, Valencia, CA, USA) following the manufacturer's protocol. We performed WGS of five isolates using an Illumina NovaSeq platform. We trimmed, quality‐checked, and aligned the sequencing reads to the Bd reference genome (strain JEL423). We identified and filtered SNPs using GATK, and conducted phylogenetic analyses in R version 4.3.1 (R Core Team 2023). To contextualise our isolates, we also analysed 22 previously published Bd genomes (Table S1). Detailed methods, including software versions, filtering parameters, and scripts, are provided in Appendix S1.

### 2.2 Microbiome

#### 2.2.1 Amplification and Sequencing

To characterise skin bacterial communities, we submitted the DNA samples extracted from swabs to the University of Michigan Microbiome Core for the amplification and sequencing of the V4 region of the 16S rRNA gene. The submitted samples included swabs from all frogs used in the transmission experiment, swabs from all individuals sampled during the field survey to collect frogs for the transmission experiment, which included individuals of the species *A. americanus* and ranids (including those not used in the experiment), and a negative control for the DNA extraction step (i.e., a clean extracted swab). Detailed amplification and sequencing protocols are provided in Appendix S1.

#### 2.2.2 Bioinformatics

For the initial processing of bacterial sequences, we imported paired‐end FASTQ files into Quantitative Insights into Microbial Ecology (QIIME 2) version 2019.1 (Bolyen et al. 2019). We utilised the DADA2 plugin to denoise, quality‐filter (truncating both forward and reverse reads to 150 base pairs to ensure maximum read quality), and to cluster sequences into amplicon sequence variants (ASVs). We assigned taxonomy to ASVs using the skicit‐learn naïve Bayes classifier and the Greengenes 13.8 reference database (DeSantis et al. 2006). To build a phylogenetic tree, we aligned sequences using the method MAFFT (Katoh et al. 2002), masking poorly aligned regions, and created the tree using the default parameters of FastTree (Price et al. 2010). We also removed sequences identified as chloroplast and mitochondria. We performed sample decontamination using the R package microDecon to identify contaminant ASVs based on those found in negative controls (McKnight et al. 2019). We removed a total of eight ASVs that were considered potential contaminants.

Additionally, we removed ASVs representing less than 0.005% (< 557) of total reads (Bokulich et al. 2013). We applied rarefaction to standardise the number of sequences across samples to 1440/sample, as rarefaction curves reached a plateau at this sequencing depth. Finally, we calculated metrics of alpha diversity (i.e., ASV richness, Faith phylogenetic diversity, and Shannon index) and beta diversity (i.e., Bray‐Curtis, Jaccard and unweighted, and weighted UniFrac distances) for each sample using the diversity core‐metrics‐phylogenetic command. After all filtering steps, the final ASV table comprised 66 samples and a total of 812 ASVs across all samples, accounting for 95,040 sequence reads. However, samples from wild frogs collected both in the field and in the laboratory during the experiment, and controls, were not included in the following statistical analyses as they were outside the scope of the study's primary objectives. In addition, an unbalanced and a small sample size of wild frogs also limited our ability to comprehensibly assess the changes in their skin bacterial communities. Therefore, the statistical analyses were conducted exclusively on data from *H. boettgeri* collected throughout the experiment (*n* = 44), which included both Bd‐negative (0 ZE) and Bd‐positive swabs (with loads varying from 2.57 ZE to 227,630 ZE; Table S2). All 16S sequence reads are deposited in the NCBI SRA database under the accession number PRJNA1156054.

#### 2.2.3 Statistical Analyses

To investigate whether our focal species *H. boettgeri* exhibits microbiome changes when infected with Bd, we assessed the changes in alpha and beta diversity of the skin bacterial communities, and conducted a LefSe (Linear Discriminant Analysis Effect Size) analysis to identify differentially abundant ASVs between Bd‐positive and Bd‐negative *H. boettgeri* hosts. We conducted all analyses in R version 4.3.1 (R Core Team 2023).

To evaluate the effect of Bd load on bacterial alpha diversity, we fitted linear and generalised linear mixed‐effects models (LMMs and GLMMs) depending on the results of Shapiro–Wilk normality tests on ASV richness, Faith phylogenetic diversity, and Shannon diversity. In all models, we used one of the alpha diversity metrics as the response variable, and Bd load (log‐transformed) as a predictor (fixed factor), and tank as a random factor. For ASV richness, we fit GLMMs using a negative binomial error distribution to account for overdispersion, using the glmer.nb function from the lme4 package version 1.1.35.2 (Bates et al. 2015). For Faith phylogenetic diversity and Shannon index, we fit LMMs with normal error distribution using the lmer function, also from the lme4 package. We visualised residual plots against model predictions to confirm appropriate error distributions. We conducted likelihood ratio tests (LRT) comparing the full models with their reduced models containing only the random effect of ‘tank’ to determine the significance of the predictor Bd load (Zuur et al. 2009).

To evaluate Bd load as a driver of change in bacterial community structure and composition based on the beta diversity metrics Bray‐Curtis, Jaccard, and UniFrac distances (weighted and unweighted), we ran PERMANOVAs (permutational multivariate analysis of variance) using the adonis2 function from the vegan package version 2.6‐8 (Oksanen et al. 2024). We specified each model with Bd load (log‐transformed) as the predictor variable, and we constrained permutations using the strata argument to account for the random effect of ‘tank’. To visualise differences in bacterial community composition and structure between samples, we conducted a principal coordinate analysis (PCoA) using the cmdscale function from the vegan package.

To identify differentially abundant ASVs between Bd‐negative and Bd‐positive *H. boettgeri* frogs, we conducted a LEfSe (Linear Discriminant Analysis Effect Size) analysis using a subset of our data encompassing negative frogs from day 0 (*n* = 12) and the surviving positive frogs on day 23 (*n* = 10) due to their highest mean loads (Table S2). We performed LEfSe using the run_lefse function from the microbiomeMarker package version 1.8.0 (Cao et al. 2022), with an LDA score (effect size) cutoff of four and Kruskal–Wallis and Wilcoxon tests with a significance level of *p* < 0.001.

## 3 Results

### 3.1 Transmission Experiment

We swabbed a total of 29 frogs, comprising the species *A. americanus* (*n* = 3), *R. clamitans* (*n* = 12), *R. pipiens* (*n* = 1), and *H. versicolor* (*n* = 13), at the Edwin S. George Reserve (ESGR). Bd DNA quantification from wild frogs revealed an overall prevalence of 34%, with Bd loads ranging from 1.22 ZE to 87.52 ZE (mean infection load of 19.68 ZE ± SD 27.76 ZE). We detected positive results for Bd in one *A. americanus* (58 ZE), nine *R. clamitans* (with a mean load of 15.71), and one *R. pipiens* (13.9 ZE). All *H. versicolor* individuals tested negative for Bd. We included the four wild individuals with the highest loads in the transmission experiment (Table S2).

**FIGURE 2:**
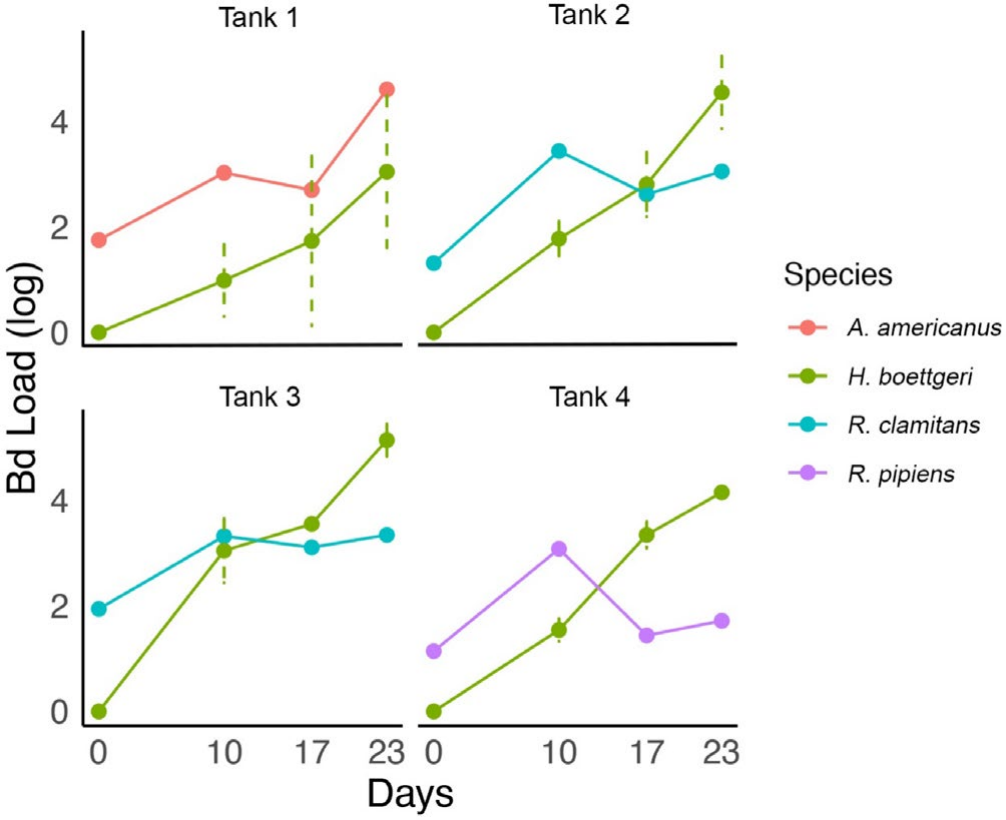
Progression of *Batrachochytrium dendrobatidis* (Bd) load in wild amphibians and *Hymenochirus boettgeri* throughout the transmission experiment. Bd load values for *H. boettgeri* represent the mean, with error bars indicating the standard deviation. All Bd load values were log‐transformed using base 10 to facilitate data visualisation and interpretation.

Following exposure to wild frogs, all *H. boettgeri* acquired Bd infections (Figure 2; Table S2). Specifically, at day 10 we were able to determine a successful transmission of Bd from wild frogs, and that this time was sufficient for an *H. boettgeri* to develop infection loads as high as 5403 ZE (Figure 2; Table S2). Mean Bd loads of *H. boettgeri* hosts in Tank 1 throughout the experiment were lower than those in the other tanks, possibly influenced by the less aquatic behaviour of the wild host (Toad) and, consequently, lower release of zoospores into the water. The mean Bd loads of *H. boettgeri* hosts in all tanks increased over time, to the point that *H. boettgeri* was unable to clear itself from the Bd infection and succumbed to the disease (Figure 2; Table S2). All wild hosts increased load considerably from day zero to day 10, followed by a reduction on day 17, and then another increase on day 23 (Figure 2; Table S2). All wild hosts died, possibly due to Bd, except for *R. pipiens* in Tank 4, which survived and showed signs of clearing the infection, with a low Bd load (52.09 ZE) on the final swab at day 23 (Table S2).

We were able to isolate Bd from seven out of twelve (58%) *H. boettgeri* , with one isolate from tank 1, one isolate from tank 2, three isolates from tank 3, and two isolates from tank 4 (Table S2). We observed that frogs sampled right after death (within 3 h) tended to have a higher probability of Bd isolation and were less prone to contamination, although this difference was not statistically significant (*p* = 0.09, Fisher's Exact Test). In fact, three of the five frogs from which we were unable to isolate Bd had died overnight and were found in an advanced stage of deterioration (body was swollen and of gelatinous appearance, the skin was also easily broken and showed fungal hyphae growing in it and a greater number of bacteria). We successfully isolated six isolates using 1% tryptone agar with antibiotics, and a seventh isolate was only possible after transferring the pieces to 1% tryptone liquid, as all of them exhibited fungal hyphal contamination. This technique allowed Bd to grow physically distant from the fungal hyphae in eight out of 13 attempts, which were subsequently transferred to 1% tryptone agar where they grew without contaminants.

**FIGURE 3:**
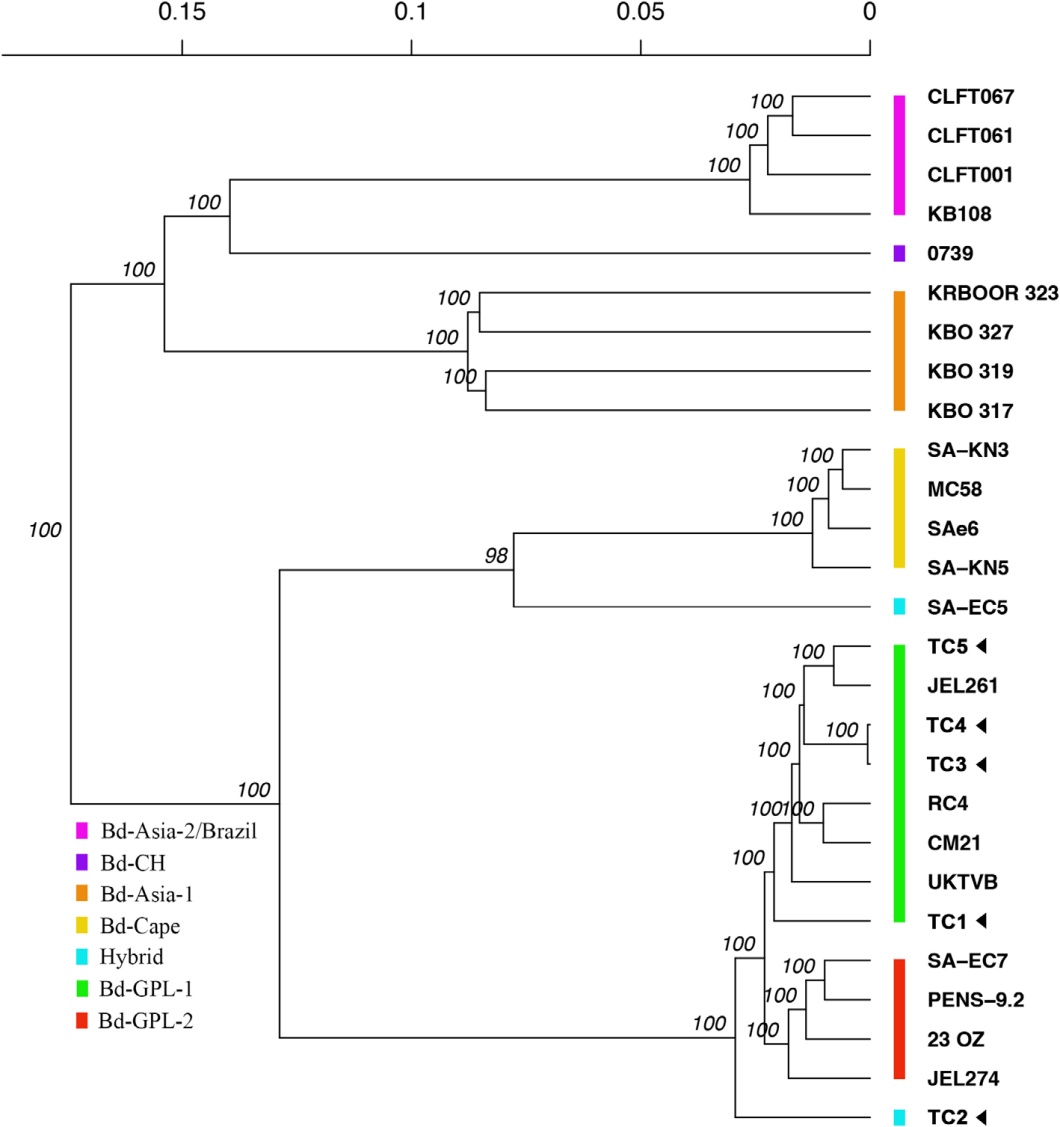
Phylogenetic tree illustrating the relationships among isolates of *Batrachochytrium dendrobatidis* (Bd), including both previously published and newly sequenced isolates for this study. Colour bars represent Bd lineages, and arrowheads indicate the newly sequenced isolates.

We obtained sequences from five Bd isolates using WGS: TC1 isolate from tank 1, TC2 isolate from tank 2, TC3 and TC4 isolates from tank 3, and TC5 isolate from tank 4 (Table S3). Our results showed that each wild host carried different genotypes of Bd, all belonging to the Bd‐GPL lineage, while the two isolates from tank 3 were identical (Figure 3). The Michigan genotypes are not closely related and showed a number of genomic differences. Like other GPL isolates, these strains have patchy heterozygosity, indicative of loss of heterozygosity (LOH) (Figure S1). Based on previous genomic and multi‐locus sequence typing data set, GPL strains have been divided into two major types, GPL‐1 and GPL‐2. In our analyses, four strains from Michigan (TC1, TC3, TC4, TC5) grouped with GPL‐1, a type that is rare outside North America and Europe. These strains shared an LOH event on the right end of scaffold 5 with other GPL‐1 strains that distinguishes them from GPL‐2 strains (Figure S2). One isolate, TC2, diverged as the most basal branch of GPL (Figure 3). TC2 had a 33% reduction in heterozygosity compared with the other Michigan strains (Figure S3), which may suggest it is a cross between two closely related GPL parents that likely share the same heterozygous genotype at most loci. In fact, prior studies have shown that throughout a global sample of the GPL, there are only two alleles per locus (James et al. 2009). We inspected the haplotypes of the Michigan strains in comparison with other GPL strains to determine whether TC2 could be a GPL‐1 × GPL‐2 recombinant, as such recombinants have been suggested from other studies of North American Bd (Byrne et al. 2022) (Figure S2). GPL‐1 is largely distinguished from GPL‐2 on the basis of specific LOH events observed on scaffolds 1 and 5 (James et al. 2015). There is little indication of a distinction between GPL‐1 and GPL‐2 on scaffold 1, but the scaffold 5 LOH event shows TC2 to have a recombinant nature. The left end of scaffold 7 shows a similar recombinant pattern for TC2. TC2 and other Michigan strains appear to be primarily disomic, based on allele frequencies (Figure S4), whereas other strains, such as JEL274 and MC58, have allele frequencies across various scaffolds that are indicative of trisomy.

### 3.2 Microbiome

**FIGURE 4:**
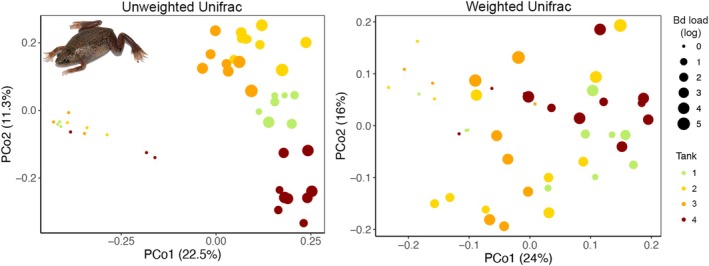
Principal coordinate analysis (PCoA) plot of Weighted and Unweighted Unifrac dissimilarities. Each point represents an individual *Hymenochirus boettgeri* , with colours indicating different tanks and point sizes representing the Bd load.

**FIGURE 5:**
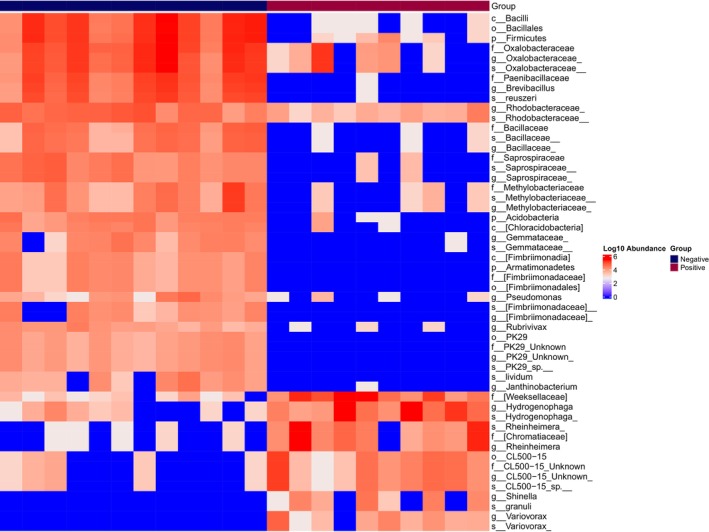
Heatmap of differentially abundant LEfSe ASVs associated with Bd infection status (negative or positive). Each row represents an individual LEfSe ASVs. Rows represent individual ASVs, and columns represent samples grouped by infection status. The colour gradient indicates log10 abundance, with red representing higher abundance and blue representing lower abundance.

We determined that Bd infection in *H. boettgeri* induced changes in the bacterial communities associated with the skin. Our models indicate that Bd load reduced the richness of ASVs, phylogenetic diversity, and Shannon index of the bacterial communities (Table 1) and also induced changes in the structure and composition of these communities (Figures 4 and S5; Table 1). Although we aimed to control for Bd genotype and wild hosts, which differed across tanks, we still observed some degree of clustering in terms of bacterial community composition and structure among the samples from the same experimental tank (Figure 4). This pattern is further supported by Figures S6 and S7, which show an increase in the number of shared bacteria between wild frogs and *H. boettgeri* over time. We also identified 52 differentially abundant ASVs in *H. boettgeri* based on the LEfSe analysis, which drove the variation between Bd‐positive (day 23) and Bd‐negative (day 0) frogs. Among these, 38 ASVs were found to be more abundant in Bd‐negative frogs, whereas 14 ASVs increased in relative abundance in Bd‐positive frogs (Table 2; Figure 5).

**TABLE 1:** Statistical metrics assessing the relationship between Bd load and diversity metrics, including alpha diversity (ASVs richness, phylogenetic diversity, Shannon index) and beta diversity (Weighted Unifrac, Unweighted Unifrac, Bray‐Curtis, Jaccard distances). Bold *p*‐values indicate statistical significance.

	Metrics	*β* Bd load	df	*χ* ^2^	*p* value
Alpha diversity	ASVs richness	−0.10	1	15.36	**< 0.001**
	Phylogenetic diversity	−0.54	1	5.21	**0.02**
	Shannon index	−0.26	1	14.21	**< 0.001**

	Metrics	df	*F*	*R* ^2^	*p* value
Beta diversity	Weighted Unifrac	1	5.49	0.12	**< 0.001**
	Unweighted Unifrac	1	8.07	0.16	**< 0.001**
	Bray‐Curtis	1	7.33	0.15	**< 0.001**
	Jaccard	1	6.86	0.14	**< 0.001**

**TABLE 2:** List of differentially abundant ASVs between Bd negative (samples from Time 0) and Bd positive (only samples from Time 3) *H. boettgeri* based on the linear discriminant analysis effect size (LEfSe) method using an LDA score cutoff of 4 and the Kruskal–Wallis and Wilcoxon tests with a significance level of *p* < 0.001.

Phylum	Class	Order	Family	Genus/Species	Enriched group	LDA score	Adjusted‐*p*
Firmicutes	Bacilli	—	—	—	Negative	5.12	6.60E−05
Firmicutes	Bacilli	Bacillales	—	—	Negative	5.11	6.60E−05
Firmicutes	—	—	—	—	Negative	5.11	9.74E−05
Proteobacteria	Betaproteobacteria	Burkholderiales	Oxalobacteraceae	—	Negative	5.02	7.43E−04
Proteobacteria	Betaproteobacteria	Burkholderiales	Oxalobacteraceae	—	Negative	4.97	9.42E−04
Proteobacteria	Betaproteobacteria	Burkholderiales	Oxalobacteraceae	—	Negative	4.97	9.42E−04
Firmicutes	Bacilli	Bacillales	Paenibacillaceae	—	Negative	4.95	4.18E−05
Firmicutes	Bacilli	Bacillales	Paenibacillaceae	*Brevibacillus*	Negative	4.92	4.18E−05
Firmicutes	Bacilli	Bacillales	Paenibacillaceae	*Brevibacillus reuszeri*	Negative	4.86	4.18E−05
Proteobacteria	Alphaproteobacteria	Rhodobacterales	Rhodobacteraceae	—	Negative	4.66	1.70E−04
Proteobacteria	Alphaproteobacteria	Rhodobacterales	Rhodobacteraceae	—	Negative	4.66	1.70E−04
Firmicutes	Bacilli	Bacillales	Bacillaceae	—	Negative	4.55	5.78E−05
Firmicutes	Bacilli	Bacillales	Bacillaceae	—	Negative	4.50	5.75E−05
Firmicutes	Bacilli	Bacillales	Bacillaceae	—	Negative	4.50	5.75E−05
Bacteroidetes	Saprospirae	Saprospirales	Saprospiraceae	—	Negative	4.49	4.94E−05
Bacteroidetes	Saprospirae	Saprospirales	Saprospiraceae	—	Negative	4.49	4.94E−05
Bacteroidetes	Saprospirae	Saprospirales	Saprospiraceae	—	Negative	4.49	4.94E−05
Proteobacteria	Alphaproteobacteria	Rhizobiales	Methylobacteriaceae	—	Negative	4.43	1.11E−04
Proteobacteria	Alphaproteobacteria	Rhizobiales	Methylobacteriaceae	—	Negative	4.42	1.11E−04
Proteobacteria	Alphaproteobacteria	Rhizobiales	Methylobacteriaceae	—	Negative	4.42	1.11E−04
Acidobacteria	—	—	—	—	Negative	4.39	7.56E−05
Acidobacteria	Chloracidobacteria	—	—	—	Negative	4.33	6.55E−05
Planctomycetes	Planctomycetia	Gemmatales	Gemmataceae	—	Negative	4.24	1.60E−04
Planctomycetes	Planctomycetia	Gemmatales	Gemmataceae	—	Negative	4.24	1.60E−04
Armatimonadetes	Fimbriimonadia	—	—	—	Negative	4.13	3.22E−05
Armatimonadetes	—	—	—	—	Negative	4.13	3.22E−05
Armatimonadetes	Fimbriimonadia	Fimbriimonadales	Fimbriimonadaceae	—	Negative	4.13	3.22E−05
Armatimonadetes	Fimbriimonadia	Fimbriimonadales	—	—	Negative	4.13	3.22E−05
Proteobacteria	Gammaproteobacteria	Pseudomonadales	Pseudomonadaceae	*Pseudomonas*	Negative	4.07	5.09E−04
Armatimonadetes	Fimbriimonadia	Fimbriimonadales	Fimbriimonadaceae	—	Negative	4.07	3.16E−04
Armatimonadetes	Fimbriimonadia	Fimbriimonadales	Fimbriimonadaceae	—	Negative	4.07	3.16E−04
Proteobacteria	Betaproteobacteria	Burkholderiales	Comamonadaceae	*Rubrivivax*	Negative	4.04	5.75E−05
Acidobacteria	Chloracidobacteria	PK29	—	—	Negative	4.03	3.22E−05
Acidobacteria	Chloracidobacteria	PK29	—	—	Negative	4.03	3.22E−05
Acidobacteria	Chloracidobacteria	PK29	—	—	Negative	4.03	3.22E−05
Acidobacteria	Chloracidobacteria	PK29	—	—	Negative	4.03	3.22E−05
Proteobacteria	Betaproteobacteria	Burkholderiales	Oxalobacteraceae	*Janthinobacterium lividum*	Negative	4.01	3.16E−04
Proteobacteria	Betaproteobacteria	Burkholderiales	Oxalobacteraceae	*Janthinobacterium*	Negative	4.01	5.53E−04
Bacteroidetes	Flavobacteriia	Flavobacteriales	Weeksellaceae		Positive	5.09	7.26E−05
Proteobacteria	Betaproteobacteria	Burkholderiales	Comamonadaceae	*Hydrogenophaga*	Positive	5.01	1.62E−04
Proteobacteria	Betaproteobacteria	Burkholderiales	Comamonadaceae	*Hydrogenophaga*	Positive	5.01	1.62E−04
Proteobacteria	Gammaproteobacteria	Alteromonadales	Chromatiaceae	*Rheinheimera*	Positive	4.87	4.93E−04
Proteobacteria	Gammaproteobacteria	Alteromonadales	Chromatiaceae		Positive	4.87	4.93E−04
Proteobacteria	Gammaproteobacteria	Alteromonadales	Chromatiaceae	*Rheinheimera*	Positive	4.87	4.93E−04
Planctomycetes	OM190	CL500‐15	—	—	Positive	4.44	6.30E−04
Planctomycetes	OM190	CL500‐15	—	—	Positive	4.44	6.30E−04
Planctomycetes	OM190	CL500‐15	—	—	Positive	4.44	6.30E−04
Planctomycetes	OM190	CL500‐15	—	—	Positive	4.44	6.30E−04
Proteobacteria	Alphaproteobacteria	Rhizobiales	Rhizobiaceae	*Shinella*	Positive	4.06	8.11E−04
Proteobacteria	Alphaproteobacteria	Rhizobiales	Rhizobiaceae	*Shinella granuli*	Positive	4.06	8.11E−04
Proteobacteria	Betaproteobacteria	Burkholderiales	Comamonadaceae	*Variovorax*	Positive	4.01	6.48E−05
Proteobacteria	Betaproteobacteria	Burkholderiales	Comamonadaceae	*Variovorax*	Positive	4.01	6.48E−05

## 4 Discussion

Our transmission experiment successfully transmitted Bd from wild amphibians to captive *H. boettgeri* , resulting in substantial Bd infection loads. This allowed for the successful isolation and whole‐genome sequencing of Bd, which revealed a high diversity of Bd genotypes at a single site, including a potential recombinant genotype between the Bd‐GPL1 and Bd‐GPL2 clades. Additionally, Bd infection led to significant shifts in the skin bacterial communities of *H. boettgeri* , with higher infection loads associated with more profound reductions in bacterial diversity and changes in community composition. These findings reinforce the use of *H. boettgeri* as a model organism to comprehensively study Bd–host interactions and the isolation of Bd from natural environments, facilitating genetic analysis to explore the distribution and genetic diversity of Bd genotypes in wild populations.

The efficient Bd isolation from adult *H. boettgeri* has a number of practical applications. For example, re‐isolating Bd after infection experiments conducted under distinct ecological conditions can offer insights into the genetic and phenotypic responses that modulate its virulence. Additionally, isolating wild Bd genotypes from experimentally infected *H. boettgeri* could complement standard isolation protocols for rare and endangered species, which involve tadpole sacrifice or adult toe amputation (Fisher et al. 2018) and frequently require large sample sizes. Indeed, the use of *H. boettgeri* could facilitate Bd isolation in regions where prevalence and/or infection loads are low. For instance, the Bd genotypes isolated in this study were the first from the state of Michigan, a region that has been sampled for years and oral disc examinations of hundreds of tadpoles failed to detect dekeratinization—a consequence of Bd infection in tadpoles (Knapp and Morgan 2006)—even with Bd prevalence exceeding 30% for some populations, as reported in this study. A greater number of Bd isolates, especially from regions where Bd remains prevalent but often at low and undetectable loads, could enhance our understanding of Bd genetic diversity, distribution patterns, and origins, while also revealing potentially undiscovered lineages. However, it is important to acknowledge that Bd isolation following a transmission experiment using *H. boettgeri* may be influenced by host‐specific factors, such as within‐host competition among Bd genotypes or differential competence of this species for particular Bd genotypes. Such processes could bias isolation outcomes, potentially underrepresenting the full diversity present in wild populations. As a result, the actual Bd diversity in a given region may be even greater than what is recovered through this method.

A similar approach to our transmission experiment was used for low‐burden infections of wild frogs in Korea, where infected adults were incubated with larvae of *Bombina orientalis* to facilitate isolation from mouthparts (Bataille et al. 2013). In that case, the resulting genotypes were identified as pivotal strains with high diversity and frequent sexual recombination, which has fueled the hypothesis that Bd is of Asian origin. In contrast, the isolation of Bd from weakly infected amphibians in this study did not reveal a novel lineage. Instead, the sequencing data for Michigan Bd revealed a high diversity of Bd‐GPL genotypes at a single site, including a potential recombinant genotype between the Bd‐GPL1 and Bd‐GPL2 clades infecting *R. clamitans* . This discovery underscores the ongoing genetic mixing of Bd‐GPL in natural environments. Possible hybrid genotypes of Bd‐GPL1 and Bd‐GPL2 have previously been reported in Pennsylvania, USA by Byrne et al. (2022), located less than 400 km from our field site. Indeed, the host species carrying the potential Bd GPL1/2 recombinant genotype in our study ( *R. clamitans* ) is the same as the one reported by Byrne et al. (2022). It is possible that the tolerance of *R. clamitans* to Bd, combined with the species' aquatic behaviour, facilitates exposure to and maintenance of different Bd genotypes, ultimately increasing the potential for hybridization. While our findings are based on a limited number of isolates from a single location, the substantial Bd diversity observed in this small geographic area suggests that Bd‐GPL may have been present on the continent longer than some estimates (O'Hanlon et al. 2018) and more in line with museum studies (Ouellet et al. 2005; Talley et al. 2015). Further research is needed to assess the evolutionary dynamics of Bd in the region and its potential impacts on amphibian populations, including whether these recombinants exhibit heightened virulence, similar to hybrid Bd‐GPL/Brazil genotypes (Greenspan et al. 2018; Carvalho, Medina, et al. 2023; Carvalho, Si, et al. 2023).

The observed shifts in the skin bacteria of *H. boettgeri* in response to Bd infection align with findings from studies on highly susceptible and endangered amphibian species (Ellison et al. 2019; Estrada et al. 2022; Buttimer et al. 2024). These changes include reductions in bacterial richness, phylogenetic diversity, and Shannon index, with more pronounced effects at higher Bd loads. Significant alterations in community structure and composition were also detected, with distinct ASVs identified between pre‐ and post‐Bd exposure. For instance, we identified ASVs from the taxonomic family Comamonadaceae that were associated with Bd infections, suggesting an increment in their relative abundances and a potential correlation with infection loads. Interestingly, ASVs from this family have previously shown an association with Bd‐exposure in other amphibian species (Jani and Briggs 2014; Walke et al. 2015; Kruger 2020; Bates et al. 2022) and have been linked to potential Bd‐inhibitory function in both tropical and temperate species (Becker, Walke, Murrill, et al. 2015; Woodhams et al. 2015; Jiménez et al. 2019; Kruger 2020). This pattern indicates that responses of *H. boettgeri* skin bacterial taxa to Bd infection also reflects fine scale patterns observed in field studies and challenge experiments focused on other amphibians. Together, these findings reinforce existing research showing that Bd infections disrupt the skin microbiome (Rebollar et al. 2020).

We also observed that experimental tanks may have explained, to a lesser extent, to the variation observed in bacterial community composition and structure. However, due to the nature of the experimental design, we were unable to tease apart whether such pattern was driven by Bd genotype or by direct transmission of bacteria from the wild host. That said, visual inspection of community changes over time suggests a convergence in skin bacterial communities across host species (wild frogs and *H. boettgeri* ), evidenced by an increase in the number of shared ASVs from day 0 to day 23. This convergence likely arose from hosts sharing environmental conditions. While our analysis centered on Bd‐induced changes in *H. boettgeri* skin bacterial communities, these preliminary results highlight the potential for cross‐host microbial exchanges that merit further investigation.

Given the observed Bd‐induced changes in the amphibian‐skin bacteria of *H. boettgeri* , this species emerges as a valuable model organism for studying the interplay among the host, their symbiotic microbes and their pathogens within an ecological and conservation context (West et al. 2019). With additional development, *H. boettgeri* could be an effective study species for exploring conservation strategies focused on microbiome interventions reducing the impact of Bd, rather than relying solely on individuals from either small captive colonies or remnant populations of endangered species.

## 5 Conclusion

Overall, this study provides a new tool for isolating Bd from wild amphibian populations by the transmission of Bd to a susceptible host in the lab, potentially revealing new lineages or genotypes and enhancing our understanding of the pathogen's genetic diversity and global distribution. The finding that *H. boettgeri* exhibits microbiome changes similar to those observed in susceptible and threatened species suggests that this model could be instrumental in developing conservation strategies, such as microbiome‐based interventions, which are crucial for mitigating the impact of Bd on endangered species (West et al. 2019). Additionally, this study uncovers a significant genotype diversity from a Bd‐enzootic amphibian assemblage, aligning with the findings of Byrne et al. (2022), which potentially contrast with the lower diversity observed in Bd‐epizootic populations from regions such as Australia, Panama, and California (O'Hanlon et al. 2018; Carvalho et al. 2024). Also, the identification of a potential recombinant genotype between the Bd‐GPL1 and Bd‐GPL2 clades underscores the ongoing genetic exchange among Bd genotypes, highlighting the importance of further research into the pathogen's genetic patterns. The existence of sexual reproduction, along with the pattern of Bd genetic diversity, emphasises the critical need to implement and continue monitoring programmes focusing on understanding the evolutionary dynamics among Bd and its amphibian hosts worldwide.

## Author Contributions

T.C.: Conceptualization, Writing – original draft, Writing – review and editing, Visualisation, Investigation, Formal analysis, Data curation, Methodology, Project administration, D.M.: Conceptualization, Writing – review and editing, Formal analysis. T.Y.J.: Conceptualization, Writing – review and editing, Supervision, Funding acquisition.

## Ethics Statement

All procedures described here were conducted at the University of Michigan Life Sciences Institute Animal Care Facilities, following protocols approved by the University's Institutional Animal Care and Use Committee (IACUC protocol PRO00009614).

## Conflicts of Interest

The authors declare no conflicts of interest.

## Supporting information


**Appendix S1:** Supporting information.

## Data Availability

The data that support the findings of this study are openly available in Zenodo at https://doi.org/10.5281/zenodo.15757221.
