# Supplementary material for: Successful Transmission and Isolation of a Fungal Pathogen From Wild Frogs to a Captive Amphibian Model Species: Fine Scale Pathogen Genetic Diversity and Infection‐Induced Changes in Skin Bacteria
Source: Environ Microbiol. 2025 Jul 3;27(7):e70136. doi: 10.1111/1462-2920.70136 (PMC12225021; doi:10.1111/1462-2920.70136)
Supplement: Supplementary file 1 — Appendix S1: Supporting information. [file EMI-27-e70136-s001.docx]

**Supplementary Information Appendix**

**Successful transmission and isolation of a fungal pathogen from wild frogs to a captive amphibian model species: fine scale pathogen genetic diversity and infection-induced changes in skin bacteria**

Tamilie Carvalho, Daniel Medina, Timothy Y. James*

^*^To whom correspondence should be addressed: tyjames@umich.edu

*Whole-genome sequencing of Bd isolates*

Whole-genome sequencing (WGS) of five isolates was conducted at the University of Michigan Advanced Genomics Core using an Illumina Novaseq instrument. The sequencing protocol followed Illumina's guidelines for 151 bp paired-end sequencing. Subsequently, BCL Convert Conversion Software v3.9.3 (Illumina) was employed to produce de-multiplexed Fastq files.

We assessed read quality metrics for each sample using FastQC version 0.11.8 (Andrews, 2010) and trimmed sequencing adapters from the reads with Trimmomatic version 0.36 (Bolger *et al.*, 2014). Finally, we assembled our reads to the Bd reference genome generated from strain JEL 423 (Broad Institute, version Jan. 2007) using BWA-MEM version 0.7.15 (Li, 2013).

To evaluate the placement of five Michigan isolates within the broader context, we acquired a set of 22 previously published globally distributed genome sequencing reads (Table S1) and aligned them with the JEL423 nuclear reference genome, following the method described above. After assembly to reference, we sorted and removed duplicate reads with Picard version 2.8.1 (Broad Institute). We realigned indels, recalibrated read quality scores and indexed reads with the Genome Analysis Toolkit suite of tools (GATK) version 4.2.2.0 (McKenna *et al.*, 2010). We identified SNP and indel variants with GATK HaplotypeCaller and performed the final joint genotyping with GATK GenotypeGVCFs. Finally, to ensure high-quality SNPs for downstream analyses, we applied variant filtration using GATK VariantFiltration with the following thresholds: variants with a Quality by Depth (QD) score below 5, a variant quality score (QUAL) below 500, a Strand Odds Ratio (SOR) above 3, or a Fisher Strand Bias (FS) above 20 were excluded. Additionally, we filtered out variants with a Mapping Quality (MQ) below 50, a total read depth (DP) outside the range of 1,000–5,000 reads, and an allele count (AN) below 40. These parameters were chosen to exclude variants likely to be artifacts or of low confidence. After filtering, we obtained a final, high-confidence panel of 274,507 SNPs for further analysis.

We imported the SNPs file into R version 4.3.1 (R Core Team, 2023) using the read.vcfR function from the vcfR package version 1.15.0 (Knaus and Grünwald, 2017), and then converted the vcfR object into a genlight object using the vcfR2genlight() function, also from the vcfR package. Finally, we constructed the phylogenetic tree using the aboot function from the poppr package version 2.9.6 (Kamvar *et al.*, 2014, 2015), employing the UPGMA (Unweighted Pair Group Method with Arithmetic Mean) algorithm alongside 100 bootstrap replicates. We used scripts written in perl (https://github.com/Michigan-Mycology/Carvalho-Transmission) to calculate allele frequencies, SNP density in sliding windows, and heterozygosity. Plotting of these metrics was done using base R and ggplot2. All Bd sequence reads are deposited in the NCBI SRA database under the accession number PRJNA1156054.

*Amplification and sequencing of bacterial communities*

The amplification was conducted for each sample using a dual-indexing sequencing PTapproach as outlined by Kozich et al. (2013). Each PCR mixture (with a total of 20 μl) comprised 2 μl of 10× AccuPrime PCR buffer II (Life Technologies), 0.15 μl of AccuPrime high-fidelity Taq DNA polymerase (catalog number 12346094; Life Technologies), 5 μl of a 4.0 μM primer set, 1 μl of DNA, and 11.85 μl of sterile double-distilled H_2_O. The PCR cycle included an initial step at 95°C for 2 minutes, followed by 30 cycles of 95°C for 20 seconds, 55°C for 15 seconds, and 72°C for 5 minutes, with a final extension at 72°C for 10 minutes. Libraries were normalized using SequalPrep Normalization Plate Kit (Life technologies catalog number A10510-01) following the manufacturer's protocol for sequential elution. The concentration of the pooled samples was assessed using the Kapa Biosystems Library Quantification kit, which is specifically designed for Illumina platforms (KapaBiosystems KK4824). Subsequently, the sizes of the amplicons in the library were analyzed utilizing the Agilent Bioanalyzer High Sensitivity DNA analysis kit (catalog number 5067-4626). The final library was composed of equimolar amounts of each sample, normalized to the pooled sample with the lowest concentration.

Sequencing was performed on an Illumina MiSeq platform using a MiSeq reagent kit v2 at 500 cycles (catalog no. MS-102-2003), following manufacturer's instructions. Libraries were prepared following Illumina's protocol for MiSeq library preparation (part number 15039740 Rev-D) for 2 nM libraries. The final loading concentration was 5 pM with a 15% PhiX spike to increase diversity. Sequencing reagents were prepared according to the 16S sequencing protocol with an Illumina MiSeq personal sequencer (16; updated protocols can be found at https://github.com/SchlossLab/MiSeq_WetLab_SOP). Custom read 1 and read 2 primers, along with index primers, were added to the reagent cartridge, and FASTQ files were generated for paired-end reads.

**Table S1.** Previously published Bd genomes used in this study.

| **NCBI SRA**  **Accession #** | **Isolate** | **Lineage** | **Locality / Country** | **Reference** |
| --- | --- | --- | --- | --- |
| SRR6375584 | KBO_317 | Bd-ASIA-1 | Gangwon-do / South Korea | O’Hanlon *et al.*, 2018 |
| SRR6375583 | KBO_319 | Bd-ASIA-1 | Gangwon-do / South Korea | O’Hanlon *et al.*, 2018 |
| SRR6375582 | KBO_327 | Bd-ASIA-1 | Gangwon-do / South Korea | O’Hanlon *et al.*, 2018 |
| SRR6375580 | KRBOOR_323 | Bd-ASIA-1 | Gangwon-do / South Korea | O’Hanlon *et al.*, 2018 |
| SRR6375524 | CLFT001 | Bd-Asia-2/Brazil | Jundiaí / Brazil | O’Hanlon *et al.*, 2018 |
| SRR6375563 | CLFT061 | Bd-Asia-2/Brazil | Pomerode / Brazil | O’Hanlon *et al.*, 2018 |
| SRR6375565 | CLFT067 | Bd-Asia-2/Brazil | Jundiaí / Brazil | O’Hanlon *et al.*, 2018 |
| SRR6375525 | KB108 | Bd-Asia-2/Brazil | Hwaseong-si / South Korea | O’Hanlon *et al.*, 2018 |
| SRR6375547 | MC58 | Bd-Cape | Limpopo / South Africa | O’Hanlon *et al.*, 2018 |
| SRR6375528 | SA-KN3 | Bd-Cape | South Africa | O’Hanlon *et al.*, 2018 |
| SRR6375526 | SA-KN5 | Bd-Cape | South Africa | O’Hanlon *et al.*, 2018 |
| SRR6375542 | SA6e | Bd-Cape | Pinetown Kwazulu / South Africa | O’Hanlon *et al.*, 2018 |
| SRR6375451 | 0739 | Bd-CH | Zurich / Switzerland | O’Hanlon *et al.*, 2018 |
| SRR6375587 | SA-EC5 | Hybrid | South Africa | O’Hanlon *et al.*, 2018 |
| SRR6375540 | 23_OZ | Bd-GPL | Nth Queensland / Australia | O’Hanlon *et al.*, 2018 |
| SRR6375458 | CM21 | Bd-GPL | Aquitaine / France | O’Hanlon *et al.*, 2018 |
| SRR6375485 | JEL261 | Bd-GPL | Quebec / Canada | O’Hanlon *et al.*, 2018 |
| SRR6375516 | JEL274 | Bd-GPL | Colorado / USA | O’Hanlon *et al.*, 2018 |
| SRR6375508 | PENS-9.2 | Bd-GPL | Spain | O’Hanlon *et al.*, 2018 |
| SRR6375496 | RC4 | Bd-GPL | Centre / France | O’Hanlon *et al.*, 2018 |
| SRR6375507 | SA-EC7 | Bd-GPL | South Africa | O’Hanlon *et al.*, 2018 |
| SRR6375445 | UKTVB | Bd-GPL | Kent / UK | O’Hanlon *et al.*, 2018 |

**Table S2.** Shown are Bd loads (*i.e*., zoospore equivalents) on days 0, 10, 17 and 23 after the beginning of the experiment, or day of death (D) during the experiment or euthanasia (E) at the end of the latter. The table also includes the outcome of the attempts to isolate Bd. Values of Bd load are not represented in a temporal sequence for *H. boettgeri*, since we did not keep track of infections at the individual level. ‘NA’ means that no attempts to isolate Bd were made.

|  | **Day 0**  **Bd load** | **Day 10**  **Bd load** | **Day 17**  **Bd load** | **Day 23**  **Bd load** | **Day of**  **death** | **Bd**  **isolated** |
| --- | --- | --- | --- | --- | --- | --- |
| **Tank 1** |  |  |  |  |  |  |
| *A. americanus* | 56.54 | 1058.47 | 499.84 | 40139.78 | 30 (D) | NA |
| *H. boettgeri* | 0 | 2.57 | 3.96 | 106.25 | 14 (D) | No |
| *H. boettgeri* | 0 | 54.83 | 742.21 | 11570.42 | 24 (D) | Yes |
| *H. boettgeri* | 0 | 6.42 |  |  | 33 (D) | No |
| Mean (*H. boettgeri*) |  | 21.27 | 373.09 | 5838.33 | 23.66 |  |
|  |  |  |  |  |  |  |
| **Tank 2** |  |  |  |  |  |  |
| *R. clamitans* | 20.64 | 2739.28 | 414.05 | 1125.89 | 36 (D) | NA |
| *H. boettgeri* | 0 | 100.62 | 361.48 | 187373.93 | 24 (D) | No |
| *H. boettgeri* | 0 | 84.86 | 3162.11 | 29254.41 | 26 (D) | No |
| *H. boettgeri* | 0 | 25.03 | 227.56 | 8001.48 | 36 (D) | Yes |
| Mean (*H. boettgeri*) |  | 70.17 | 1250.38 | 74876.61 | 29 |  |
|  |  |  |  |  |  |  |
| **Tank 3** |  |  |  |  |  |  |
| *R. clamitans* | 87.52 | 2093.74 | 1281.94 | 2203.46 | 34 (D) | NA |
| *H. boettgeri* | 0 | 5403.81 | 3522.19 | 227630.06 | 16 (E) | Yes |
| *H. boettgeri* | 0 | 667.24 | 3602.01 | 83423.14 | 21 (D) | Yes |
| *H. boettgeri* | 0 | 379.37 |  |  | 25 (D) | Yes |
| Mean (*H. boettgeri*) |  | 2150.14 | 3562.10 | 155526.6 | 21 |  |
|  |  |  |  |  |  |  |
| **Tank 4** |  |  |  |  |  |  |
| *R. pipiens* | 13.89 | 1201.11 | 27.53 | 52.09 | 28 (E) | NA |
| *H. boettgeri* | 0 | 42.65 | 2102.27 | 14694.81 | 26 (D) | Yes |
| *H. boettgeri* | 0 | 50.53 | 1271.97 | 13304.91 | 27 (D) | Yes |
| *H. boettgeri* | 0 | 19.95 | 4033.46 | 14406.43 | 28 (E) | No |
| Mean (*H. boettgeri*) |  | 37.71 | 2469.23 | 14135.38 | 27 |  |

**Table S3.** Bd isolates from Michigan with whole-genome sequences generated in this study.

| **Isolate** | **Accession number** | **Lineage** | **# Reads** | **% Reads** | **NCBI SRA Accession** |
| --- | --- | --- | --- | --- | --- |
| TC1 | MICH: CZEUM 351281 | Bd-GPL | 78,413,377 | 0.64 | SRR30524032 |
| TC2 | MICH: CZEUM 351282 | Bd-GPL | 65,900,162 | 0.537 | SRR30524031 |
| TC3 | MICH: CZEUM 351283 | Bd-GPL | 71,175,947 | 0.583 | SRR30524030 |
| TC4 | MICH: CZEUM 351285 | Bd-GPL | 61,431,347 | 0.5 | SRR30524029 |
| TC5 | MICH: CZEUM 351286 | Bd-GPL | 67,152,038 | 0.547 | SRR30524028 |

**
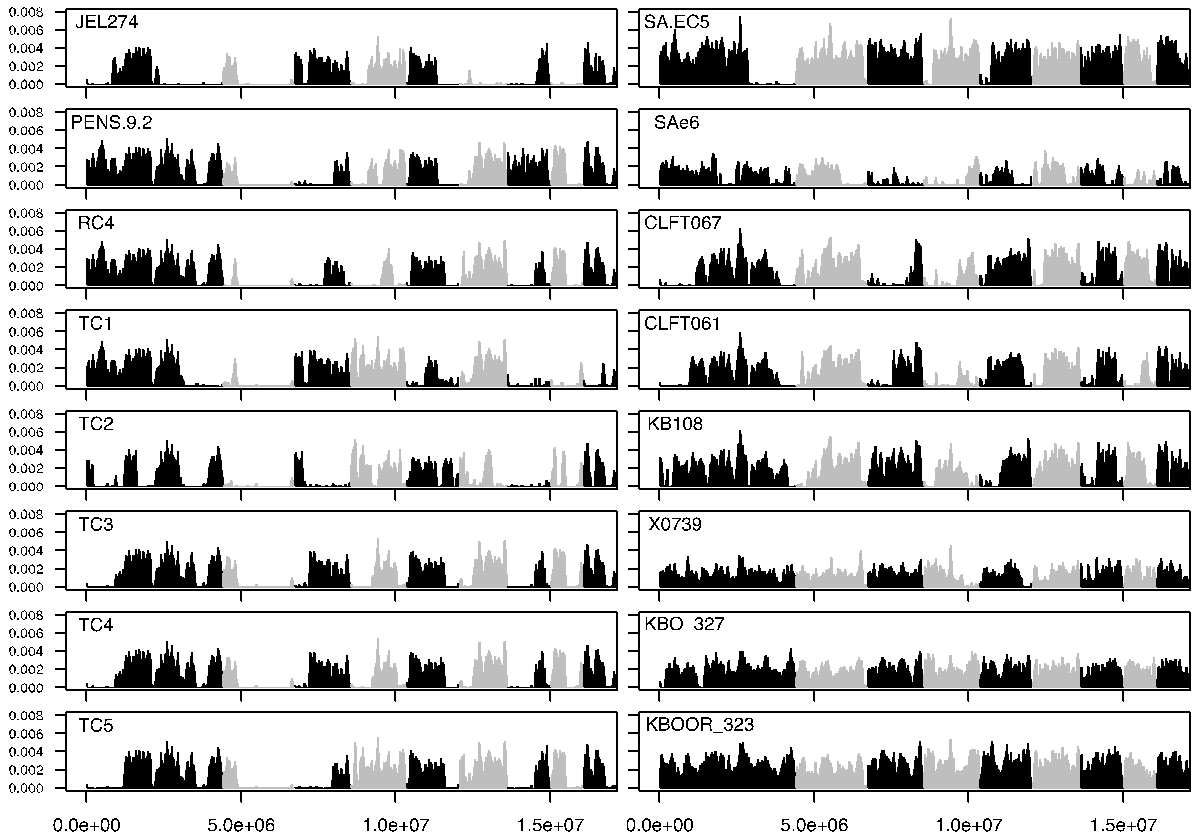
**

**Figure S1.** Landscape plots of heterozygosity of Bd strains plotted along a sliding window of 50 kb size and step size of 10 kb. Shown is mean heterozygosity along the 9 largest scaffolds with scaffolds indicated by alternative shading.

**Figure S2.** Haplotype blocks compared between GPL-1 strains and GPL-2 strains reveal unusual structure in putative hybrid TC2. Shown are the largest 17 scaffolds. Blocks were computed from the modal genotype in windows of size 20 kb. Grey regions indicate heterozygous regions. Red regions indicate homozygous regions present in strain JEL274. Blue regions are homozygous regions not found in JEL274. Green arrows indicate regions that distinguish GPL-1 from GPL-2 and are indicative of recombination in TC2.

**Figure S3.** Number of total heterozygous positions across the five Michigan strains indicates lower heterozygosity of TC2.

**Figure S4.** Allele frequency histograms for the 9 largest scaffolds. Most scaffolds in TC strains show a single peak indicative of a disomic chromosomal number.

**
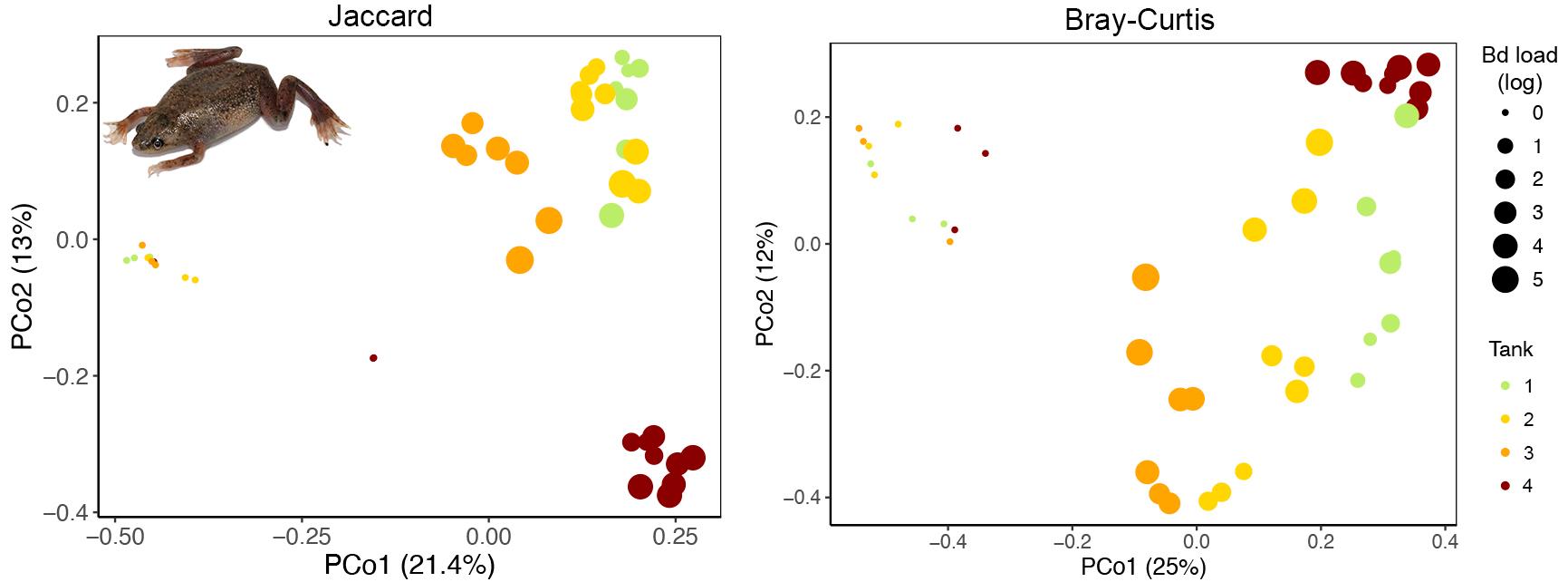
**

**Figure S5.** Principal coordinate analysis (PCoA) plot of Jaccard and Bray-Curtis dissimilarities. Each point represents an individual frog, with colors indicating different tanks and point sizes representing the Bd load.

**
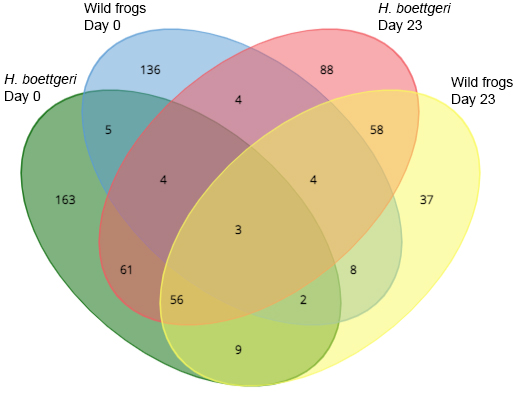
**

**Figure S6.** Venn diagram showing the increase in the number of ASVs shared between *Hymenochirus boettgeri* and wild frogs over time (comparison between Day 0 and Day 23). Samples from all *H. boettgeri* individuals were grouped together, as were samples from all wild frogs.

**
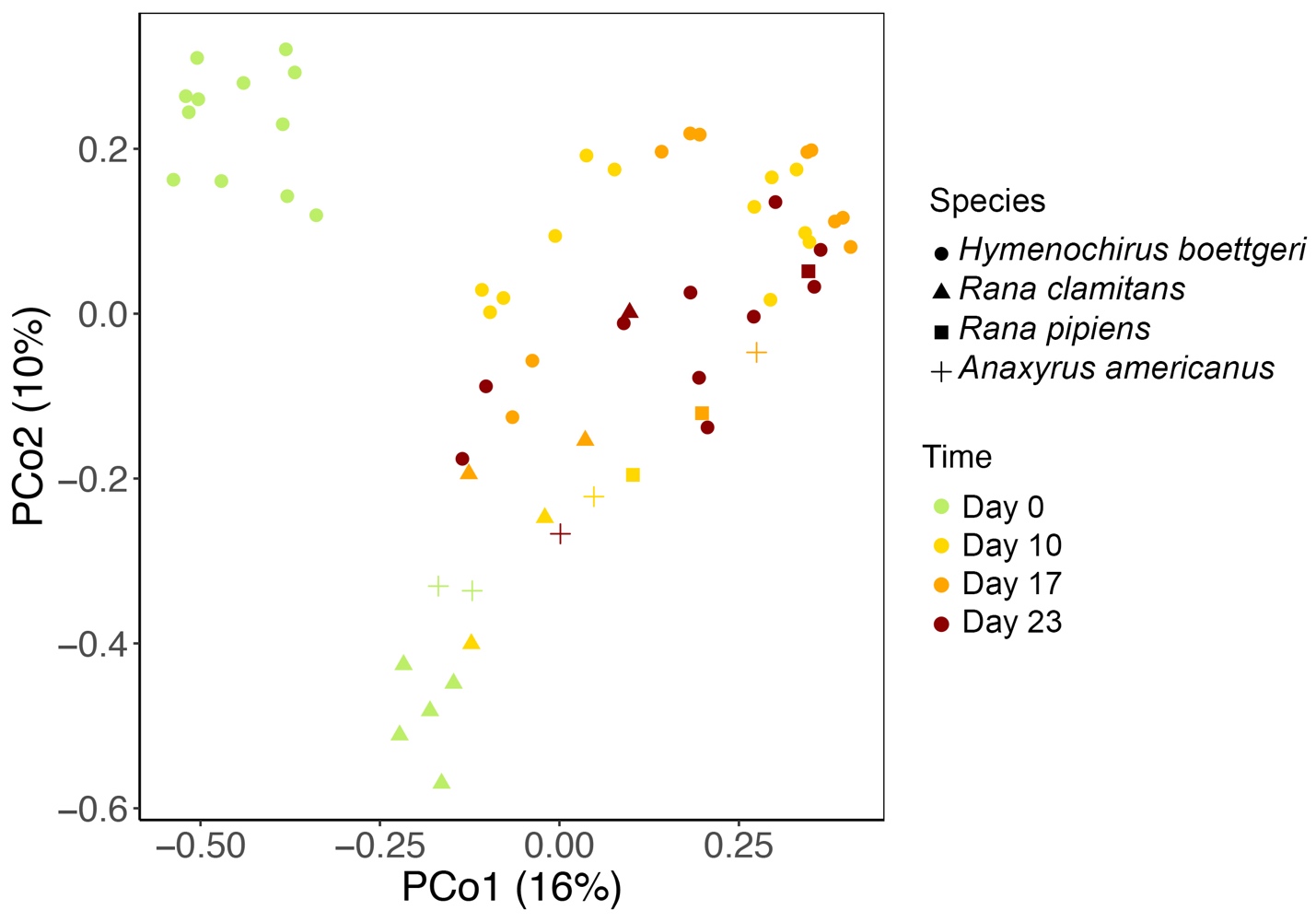
**

**Figure S7.** Principal Coordinates Analysis (PCoA) based on Bray-Curtis dissimilarity showing bacterial community composition across amphibian host species over time. Each point represents the skin microbiome of an individual frog. Colors indicate the sampling day and shapes represent different host species. At Day 0, *H. boettgeri* samples clustered separately from wild species, which were more similar to one another. From Day 10 through Day 23, all host groups showed increasing similarity in bacterial community composition, suggesting microbial convergence likely driven by shared environmental conditions. One *Rana pipiens* sample from Day 0 and one *Rana clamitans* sample from Day 23 were excluded due to insufficient sequencing reads.

**References**

Andrews, S. (2010) FastQC: a quality control tool for high throughput sequence data.

Bolger, A.M., Lohse, M., and Usadel, B. (2014) Trimmomatic: a flexible trimmer for Illumina sequence data. *Bioinformatics* **30**: 2114–2120.

Kamvar, Z.N., Brooks, J.C., and Grünwald, N.J. (2015) Novel R tools for analysis of genome-wide population genetic data with emphasis on clonality. *Front Genet* **6**:.

Kamvar, Z.N., Tabima, J.F., and Grünwald, N.J. (2014) Poppr: an R package for genetic analysis of populations with clonal, partially clonal, and/or sexual reproduction. *PeerJ* **2**: e281.

Knaus, B.J. and Grünwald, N.J. (2017) vcfr: a package to manipulate and visualize variant call format data in R. *Molecular Ecology Resources* **17**: 44–53.

Kozich, J.J., Westcott, S.L., Baxter, N.T., Highlander, S.K., and Schloss, P.D. (2013) Development of a Dual-Index Sequencing Strategy and Curation Pipeline for Analyzing Amplicon Sequence Data on the MiSeq Illumina Sequencing Platform. *Applied and Environmental Microbiology* **79**: 5112–5120.

Li, H. (2013) Aligning sequence reads, clone sequences and assembly contigs with BWA-MEM.

McKenna, A., Hanna, M., Banks, E., Sivachenko, A., Cibulskis, K., Kernytsky, A., et al. (2010) The Genome Analysis Toolkit: A MapReduce framework for analyzing next-generation DNA sequencing data. *Genome Res* **20**: 1297–1303.

O’Hanlon, S.J., Rieux, A., Farrer, R.A., Rosa, G.M., Waldman, B., Bataille, A., et al. (2018) Recent Asian origin of chytrid fungi causing global amphibian declines. *Science* **360**: 621–627.

R Core Team (2023) R: A Language and Environment for Statistical Computing.
